# Validation of Zulu Watch against Polysomnography and Actigraphy for On-Wrist Sleep-Wake Determination and Sleep-Depth Estimation

**DOI:** 10.3390/s21010076

**Published:** 2020-12-25

**Authors:** Jaime K. Devine, Evan D. Chinoy, Rachel R. Markwald, Lindsay P. Schwartz, Steven R. Hursh

**Affiliations:** 1 Institutes for Behavior Resources, Inc., Baltimore, MD 21218, USA; lpschwartz@ibrinc.org (L.P.S.); shursh@ibrinc.org (S.R.H.); 2 Sleep, Tactical Efficiency, and Endurance Laboratory, Warfighter Performance Department, Naval Health Research Center, San Diego, CA 92106, USA; evan.d.chinoy.ctr@mail.mil (E.D.C.); Rachel.r.markwald.civ@mail.mil (R.R.M.); 3 Leidos, Inc., San Diego, CA 92106, USA

**Keywords:** actigraphy, sleep tracking, validation, wearable device, sleep scoring, longitudinal data collection, real-world sleep tracking

## Abstract

Traditional measures of sleep or commercial wearables may not be ideal for use in operational environments. The Zulu watch is a commercial sleep-tracking device designed to collect longitudinal sleep data in real-world environments. Laboratory testing is the initial step towards validating a device for real-world sleep evaluation; therefore, the Zulu watch was tested against the gold-standard polysomnography (PSG) and actigraphy. Eight healthy, young adult participants wore a Zulu watch and Actiwatch simultaneously over a 3-day laboratory PSG sleep study. The accuracy, sensitivity, and specificity of epoch-by-epoch data were tested against PSG and actigraphy. Sleep summary statistics were compared using paired samples *t-*tests, intraclass correlation coefficients, and Bland–Altman plots. Compared with either PSG or actigraphy, both the accuracy and sensitivity for Zulu watch sleep-wake determination were >90%, while the specificity was low (~26% vs. PSG, ~33% vs. actigraphy). The accuracy for sleep scoring vs. PSG was ~87% for interrupted sleep, ~52% for light sleep, and ~49% for deep sleep. The Zulu watch showed mixed results but performed well in determining total sleep time, sleep efficiency, sleep onset, and final awakening in healthy adults compared with PSG or actigraphy. The next step will be to test the Zulu watch’s ability to evaluate sleep in industrial operations.

## 1. Introduction

Sleep impacts almost every domain of human function: from mood to job performance to physical health and mortality [1,2,3]. Whether the goal of a monitoring system in the 4.0 Era is to improve personal well-being or to overhaul industry infrastructure, controlling for sleep as an intervening variable can only improve the model. Sleep researchers have been tracking sleep using wrist-worn actigraphy since the late 1970s [4]; recently, commercial wrist-worn wearable devices have begun to feature sleep-tracking technology as well. The Sleep Research Society recently published a white paper outlining the barriers and opportunities for using wearables in sleep and circadian science [5]. This paper identified poor validation of wearable technology as the primary barrier inhibiting the use of wearables in sleep and circadian science. Device validation is also important for industrial or clinical research applications.

The first step towards validating a wearable device begins with laboratory testing against the gold-standard measurements of sleep; i.e., polysomnography (PSG) and research-grade actigraphy. Measuring sleep with the gold-standard technique polysomnography (PSG) provides high-quality data, but it has many requirements, such as an overnight visit to a sleep laboratory, the application of multiple physiological recordings (on the scalp, face, and body), and scoring of recordings by an expert trained in the American Academy of Sleep Medicine (AASM) guidelines [6]. The output from the PSG data are then condensed into summary measures like time in bed (TIB), total sleep time (TST), sleep efficiency (SE; the ratio of TST over TIB), and sleep architecture. The sleep architecture data are used to further categorize the night into three non-rapid eye movement (NREM) and one rapid eye movement (REM) sleep stages, each with distinct patterns of physiology and behavior that indicate “sleep depth”. Starting with NREM, the sleep stages cycle throughout the night, each playing a critical role in the maintenance of the brain and body to promote optimal health and functioning [7].

In contrast to PSG, actigraphy can measure TIB, TST, SE, and sleep behaviors like napping, but it only categorizes sleep and wake as binary outcomes and therefore does not provide an estimate of sleep depth. Wrist-worn actigraphy devices can automatically determine sleep from activity levels using validated algorithms, but data must first be downloaded from the device to specialized software. Importantly, traditional actigraphy caters to healthy adult populations with regular sleep patterns. Its application to operational environments requires significant additional data processing. While the practice parameters for the clinical or research use of actigraphy in healthy individuals, sleep-disordered populations, or military operational contexts have been published [8,9,10], these guidelines still encourage hand scoring of data to achieve the highest levels of accuracy, and there is no resolute consensus on the scoring methodology [11,12].

Many commercial wearables have been tested against PSG or actigraphy for sleep-wake determination with mixed, but promising, results [13,14,15,16,17,18]. The measurement of wrist activity by accelerometry alone cannot estimate sleep stages or NREM–REM sleep stage cycles, but it can identify bouts of immobility which are known to correspond to periods of restful sleep that include NREM and REM [19,20]. Many wearables also incorporate other sensors, such as heart rate, oxygen saturation, or peripheral arterial tone, to supplement wrist activity data [20,21]. These devices can be used to track shorter sleep episodes like napping and can provide an estimate of sleep depth similar to sleep stages. However, while the devices collect accelerometry and other biometric data, sleep scoring is done using algorithms on a companion mobile app or software platform [14,22]. This necessity restricts the ability of devices to log sleep episodes in the absence of wireless connectivity to download data, and it can generate concerns about privacy among researchers and consumers alike [23,24]. Furthermore, commercial sleep trackers are designed to provide direct feedback to the consumer about their sleep, a feature that may not always be desirable for research study purposes. Lastly, the battery life of most research-grade actigraphy devices is only a few weeks and for most commercial devices only a few days, meaning that changes to sleep behavior over the long term cannot be continually observed. This constitutes a limitation to any analyses that strive to investigate changes in sleep over time or the cumulative effects of sleep behavior patterns. Frequently changing the battery or recharging the device may be a deterrent to wearing the device or may not be feasible in all populations and settings. The number of active sensors or features like automatic syncing can affect the longevity of a charge, creating a trade-off between what data can be collected and the time-frame of the collection period. Battery life, therefore, is an important limiting factor when choosing a device for research, clinical, or personal measurement of sleep [12,25].

Sleep is a complex psychosocial behavior in addition to being a neurobiological phenomenon. Developing a wearable that can account for variability in sleep behaviors across individuals and over time as well as providing an estimate of sleep depth would bridge the gap between laboratory and epidemiological concepts of sleep. Bridging this gap does not necessarily require significant innovation to hardware or algorithms, but does require addressing the specific needs of real-world applications. The Zulu watch (Institutes for Behavior Resources, Baltimore, MD, USA) is a novel commercial sleep-tracking device designed specifically for operational environments. The Zulu watch measures sleep duration and estimates sleep depth based on wrist movement using a tri-axial accelerometer and on-wrist detection using a galvanic sensor. It scores sleep on-wrist rather than through a software or cloud application. It features the option to either provide sleep feedback directly to the user or to export sleep data for research purposes. The watch features a light sensor, on-demand heart rate, and off-wrist detection, and can store data on up to 80 sleep intervals and 7 days’ worth of 2-min epoch-by-epoch (EBE) data at a time, which can be extracted to .csv files for analysis. The battery of the Zulu watch can last up to one year with all these features active. Older sleep interval and EBE data are automatically overwritten by the newly collected data to provide for continuous longitudinal measurement of sleep. The Zulu watch uses sleep history data to compute a real-time estimate of sleep debt when in feedback mode. Researchers may extract this data intermittently per their study design and return the watch to the wearer immediately. The long battery life and ability of the Zulu watch to store up to 80 sleep intervals on-wrist means that researchers can collect data over a longer period of time than the 14- to 60-day range typical of research actigraphy devices. The Zulu watch can also detect multiple sleep periods per day, identify naps as short as 20 min, and can be worn as a normal wristwatch to tell time. The Zulu watch was designed as a sleep-tracking device that could serve either as a commercial wearable or as a research-grade actigraph for use in operational environments where sleep opportunities may be limited and the quality of the sleep environment may be compromised.

While the Zulu watch is intended for longitudinal measurement of sleep in real-world environments, laboratory testing against the gold standards under controlled conditions is an important initial step towards establishing the performance of a sleep-tracking device [5]. Therefore, the aims of this validation study were to (1) test the agreement between the sleep-tracking algorithm in the Zulu watch and both gold-standard laboratory PSG and research-grade actigraphy for sleep summary outcome measures and EBE sleep-wake determination; and (2) test the agreement between the Zulu watch’s sleep-depth scoring and PSG sleep staging.

## 2. Materials and Methods

### 2.1. Participants

Eight (8) participants wore both a Zulu watch and Actiwatch 2 (Philips Respironics, Murrysville, PA, USA) continuously over the course of a consecutive 3-day PSG sleep study. Participants were all healthy young adults, consisting of four men and four women who met the following inclusion criteria based on a self-report medical history questionnaire: age, 18–35 years (30.4 ± 3.2 years; mean ± SD); average nightly sleep duration, 6–9 h; body mass index, 18.5–29.9 kg/m^2^; no diagnosed sleep, mental health, or medical disorders; no use of any sleep medications or nicotine in the previous month; no use of illegal drugs in the previous 6 months; women who are not pregnant; and no travel across more than one time zone or any shift work in the previous month. These criteria are standard for inclusion in laboratory PSG sleep research studies in healthy adults. See Chinoy et al. for additional study participant screening details [18].

The study, which took place at the Naval Health Research Center (NHRC), was approved by the NHRC Institutional Review Board, and was conducted in accordance with the Declaration of Helsinki. Participants signed informed consent prior to the study and were compensated for their participation.

### 2.2. Procedure

Prior to study initiation, participants kept a consistent self-selected habitual 8-h TIB sleep schedule for 4 nights. Deviations (earlier or later) in bed and wake times of up to 30 min from the sleep schedule were allowed if the 8-h TIB requirement was upheld each night. Adherence to the pre-study sleep schedule was verified by sleep diary and actigraphy data.

Participants arrived at the laboratory each evening and were provided with an 8-h TIB sleep opportunity at the same time each night, based on their habitual bed and wake times as calculated from the average of their 4-night self-selected pre-study sleep schedule. Individual sound attenuated and lighting-controlled research bedrooms were used for sleep testing. Participants left the laboratory during the day but were instructed to wear both devices continuously at home, except for during showers or rigorous activities during which the watches could have been damaged. Beginning with the pre-study period and throughout the study, participants were instructed to not consume any caffeine or alcohol or nap. At the start of each evening, participants signed an attestation form verifying that they adhered to the study protocol and passed an alcohol breathalyzer test to verify sobriety. Room lighting was set to ~150 lux, and participants were observed continuously while in the laboratory and engaged in sedentary activities before bedtime. Participants arrived at the laboratory 2.5 h prior to bedtime on study nights 1 and 3, and 4.5 h prior to bedtime on study night 2. Application of the PSG electrodes began approximately 2 h before the participants’ bedtimes. Participants left the laboratory approximately 1 h after wake time on study night 1, and 3 h after wake time on study nights 2 and 3. See Chinoy et al. for additional study testing protocol details [18].

### 2.3. PSG Recording

PSG included electroencephalographic (EEG: F3-M2, F4-M1, C3-M2, C4-M1, O1-M2, and O2-M1), electromyographic (EMG: mentalis chin muscle), left and right electrocardiographic, and electrooculographic (EOG: E1-M2 and E2-M1) recordings performed according to AASM guidelines [6]. PSG data were sampled at 256 Hz; EEG and EOG were filtered at 0.3–35 Hz; and EMG was filtered at 10–100 Hz. Impedances were verified to be ≤10 kΩ at the start of the PSG recordings. The following standard sleep parameters were calculated for the purposes of this study: TIB, in minutes, as time from lights out to lights on; TST in minutes; and SE as a percentage (TST/TIB*100). Sleep stages (wake, N1, N2, slow-wave sleep (SWS), and REM) were scored by registered polysomnographic technologists (RPSGTs) in 30-s epochs, according to standard criteria [6].

### 2.4. Actigraphy

An Actiwatch 2 device was worn on the participant’s non-dominant wrist (same wrist as the Zulu watch) throughout the study period. Activity data were collected in 30-s epochs. Sleep interval start and end times were manually set by a researcher using Actiware software (version 6.0.9) in accordance with the PSG recording period. Sleep–wake determination and the TIB, TST, and SE for each sleep interval using EBE data were computed by the Actiware 6.0.9 scoring algorithm using the medium sensitivity threshold.

### 2.5. Automatic Sleep Determination and Sleep Scoring by the Zulu Watch

The Zulu watch hardware device collects activity data in 2-min epochs and automatically scores TIB and SE on-wrist, based on a proprietary algorithm for sleep-wake determination. Devices were programmed to detect multiple sleep episodes per day; the minimum sleep detection was set at 20 min. Data were then exported as the scored sleep interval information and as 2-min EBE data for the duration of the study period. TST was calculated as TIB*SE to determine the number of minutes during the sleep interval in which the Zulu watch determined that sleep was occurring. Epoch data are scored as on-wrist “On” or off-wrist “Off.” Epochs are scored in a separate data column as 0 for periods of wake, 1 for restless or interrupted sleep, 2 for light sleep, and 3 for deep sleep. The Zulu watch uses a propriety algorithm to estimate sleep depth, using only motion and on-wrist detection, and cannot differentiate between sleep stages. Zulu watch sleep-depth scoring should be considered an estimation of locomotor inactivity rather than an estimate of neurophysiological sleep architecture.

### 2.6. Analyses

All data were analyzed using Excel 2013 and Stata/MP 15 software. Statistical significance was set at *p* < 0.05. The statistical plan for the validation of the Zulu watch against PSG and actigraphy was guided by the recommendations set forth by the Sleep Research Society for the validation of commercial sleep wearables against the gold-standard PSG and research-grade actigraphy [5]. One participant’s third-night data were excluded because the Zulu watch indicated that it was off-wrist during the study night. Another participant’s second-night data were excluded because of technical issues collecting the PSG data.

For sleep-wake determination, all sleep epochs were coded as 1, and all wake epochs were scored as 0 for PSG, Actiwatch 2, and Zulu watch data. PSG and Actiwatch 30-s epochs were recalculated into 2-min epochs by averaging the binary sleep scores into 2-min bins and rounding up to the nearest integer. Using binary sleep-wake scores, Zulu watch EBE data were compared against the standard measures (PSG or Actiwatch 2) by subtracting the Zulu watch sleep scores from the corresponding standard measure to determine the agreement for individual epochs. Sensitivity, specificity, and accuracy were then computed for the total recording period. True positives were considered all epochs wherein a Zulu watch score indicates sleep and was in agreement with the standard measures’ (PSG or Actiwatch 2) epochs indicating sleep, while true negatives were considered epochs where the Zulu watch scores indicating wake were in agreement with the PSG or Actiwatch 2 epochs indicating wake. Accuracy ((true positives + true negatives)/all epochs) was calculated as the proportion of all epochs wherein the Zulu watch scoring was in agreement with the standard measures over the total recorded epochs. Sensitivity (true positives/(true positives + false negatives)) was calculated as the proportion of all epochs identified as sleep by the Zulu watch over all epochs identified as sleep by the standard measures, and specificity (true negatives/(true negatives + false positives)) was calculated as the proportion of all epochs identified as wake by the Zulu watch over all epochs identified as wake by the standard measures. Accuracy, sensitivity, and specificity were also calculated for Actiwatch 2 compared against PSG. Differences in accuracy, sensitivity, and specificity between the Actiwatch 2 and Zulu watches compared against PSG were evaluated using paired samples *t-*tests, and differences across all three sets of comparisons were evaluated using one-way analysis of variance.

**Figure 1:**
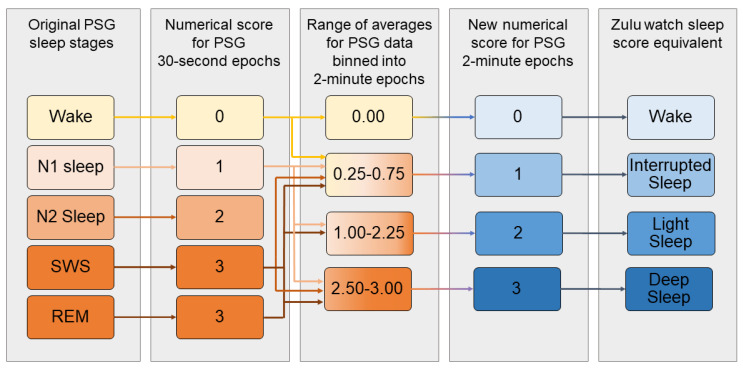
Recoding the polysomnography (PSG) sleep stages from 30-s epochs into 2-min Zulu watch sleep score equivalents. REM, rapid eye movement; SWS, slow-wave sleep.

The Zulu watch sleep-depth scores were not considered equivalent to the PSG sleep stages. The Zulu sleep scores indicate depth-of-sleep via wrist inactivity rather than neurophysiological brain activity, and the data are collected in 2 min epochs instead of 30-s epochs. However, the ability of the Zulu watch to detect periods of inactivity that overlap with periods of restorative sleep was explored by converting the PSG sleep stages into a numerical code and comparing them against the Zulu watch sleep scores. The PSG scores were recalculated into 2-min bins by averaging the sleep-staging scores from 30-s epochs into 2-min bins and recoding these bins to approximate the Zulu watch scores. Figure 1 shows the logic for recoding the PSG stages and the Zulu watch sleep scores developed for this study. It should be noted that the numerical scores for the PSG 30-s epochs range between 0 and 3 but are not considered directly equivalent to the Zulu watch sleep scores (0–3). Bins with an average of 0.0 indicated wakefulness for the entire period and were considered equivalent to a Zulu watch score of 0 (wake). Bins with averages between 0.25 and 0.75 (indicating a mixture of epochs scored as sleep and wake within a 2-min period) were considered equivalent to interrupted sleep, or a Zulu score of 1. Light sleep (Zulu watch score of 2) was defined to be any 2-min bins with an average between 1 and 2.25. Deep sleep (Zulu watch score of 3) was defined as any 2-min bins with an average ≥2.5. Zulu watch data were then compared bin by bin with PSG by subtracting the Zulu watch sleep scores from the corresponding PSG score to determine the agreement for individual epochs. The accuracy, sensitivity, and specificity were calculated as previously described for the proportion of all bins wherein the Zulu watch scoring was in agreement with the PSG bins for interrupted sleep, light sleep, or deep sleep.

Sleep summary statistics (TIB, TST, SE, time of sleep onset, and time of final awakening) were calculated over the PSG recording period and were extracted from Actiware or Zulu watch sleep interval export data for the Actiwatch 2 and Zulu watches, respectively. Zulu watches detect periods of wakefulness during sleep episodes but do not provide an on-wrist report of sleep-onset latency (SOL), wake after sleep onset, or number of awakenings, so these measures could not be compared against PSG or actigraphy. Differences between PSG and the Zulu watch and Actiwatch 2 watch sleep summary statistics were examined using paired samples *t-*tests. Agreement between the summary statistics was evaluated using single rater, two-way random effects intraclass correlation coefficients (ICCs) with absolute agreement. The ICC values were classified as poor (<0.50), moderate (0.50–0.75), good (0.75–0.90), or excellent (>0.90) based on established guidelines [26]. Differences in sleep summary statistics from the Zulu watch and Actiwatch 2 compared with the gold-standard PSG were additionally visualized using Bland–Altman plots. Limits of agreement were computed (mean difference ± 1.96 SD) to indicate the range in which the differences between the two measures would occur with 95% probability [27].

Because Zulu watches can detect multiple sleep episodes as short as 20 min per day, there were instances when a participant’s Zulu watch logged sleep intervals occurring outside of the PSG recording period. The timing and duration of extra sleep intervals were compared with the watch’s off-wrist detection record and the timing of study procedures to determine whether the logged interval most likely represented a time when the watch was off-wrist, a time when the study procedures required the participant to restrict movement (such as when placing electrodes on the scalp for PSG or completing a cognitive test), or a possible nap.

## 3. Results

### 3.1. Zulu Watch Sleep-Wake Determination Compared with PSG and Actigraphy

**Figure 2:**
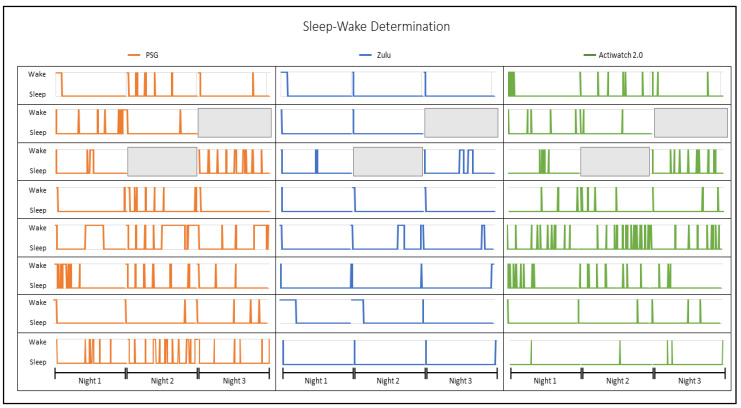
Comparison of the participants’ epoch-by-epoch sleep-wake hypnograms by polysomnography (PSG), Zulu watch, and actigraphy. A three-panel comparison of the PSG, Zulu watch, and Actiwatch 2 sleep-wake determination across all study nights by participant. Epochs are shown in study days and graphed along the *x*-axis. Sleep versus wake determination is plotted in orange (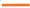) for PSG, blue (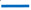) for the corresponding Zulu watch data, and green (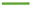) for the corresponding Actiwatch 2 data. Missing nights of data are indicated by gray blocks (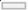).

The accuracy, sensitivity, and specificity between the sleep measurement methods are summarized in Table 1. EBE data (shown as hypnograms in Figure 2) were compared in 2-min bins across all measures. Compared with PSG or actigraphy, the accuracy for the determination of sleep by the Zulu watches was high, as was the sensitivity to detect sleep epochs. However, Zulu watches showed low specificity for identifying epochs of wake during a sleep interval. For comparison, the accuracy, sensitivity, and specificity were also computed for the Actiwatch 2 compared with PSG from this dataset (see Table 1), which also had a high accuracy and sensitivity but low specificity. Paired samples *t-*tests indicated a trend for Zulu watches to have higher percentages for specificity versus PSG than the Actiwatch 2 (*t* = 1.92, *p* = 0.06). The accuracy and sensitivity versus PSG were comparable between the Zulu watch and Actiwatch 2 (all *p* > 0.42).

### 3.2. Zulu Watch Sleep-Depth Scoring Compared With PSG

**Figure 3:**
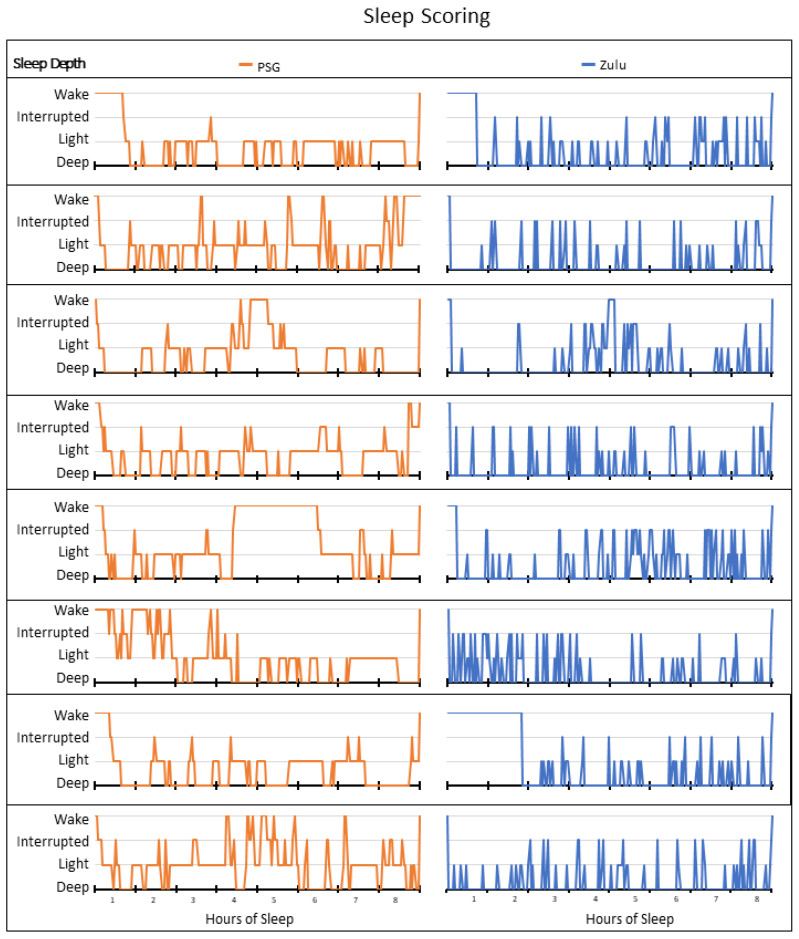
Comparison of the participants’ epoch-by-epoch sleep-wake hypnograms by polysomnography (PSG) and Zulu watch. A two-panel comparison of the paired PSG and Zulu watch sleep-scoring data from the first study night for each participant. Only the first night is depicted in order to improve readability. Epochs are graphed along the *x*-axis and shown in hours of sleep over the study night. Sleep stages are plotted in orange (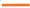) for PSG and blue (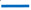) for the corresponding Zulu watch data.

The accuracy, sensitivity, and specificity for the determination of interrupted, light, and deep sleep are summarized in Table 2. EBE data were compared by 2-min bins (example sleep-depth hypnograms are shown in Figure 3). The Zulu watches showed a high accuracy for the determination of interrupted sleep and moderate accuracy for the determination of light sleep and deep sleep. The Zulu watch was particularly sensitive for detecting deep sleep compared with interrupted sleep, light sleep, or wake.

### 3.3. Zulu Watch Determination of Sleep Summary Statistics Compared with PSG and Actigraphy

Sleep summary statistics as determined by PSG, Actiwatch 2, and Zulu watch are summarized in Table 3. The TIB was set by the researchers at a constant of 480 min for all participants across all nights in the PSG and Actiwatch 2 datasets but was automatically determined by the Zulu watch. The lack of variability in TIB between the PSG and Actiwatch 2 measurements prohibited statistical testing, as we have noted. Paired samples *t-*test analyses indicated agreement between the PSG and Zulu watch measures of TST, time of sleep onset, and time of final awakening, but a lack of agreement between the PSG and Zulu watch measures of SE. Actiwatch 2 and Zulu watch measures of SE, time of sleep onset, and time of final awakening were in agreement, but paired samples *t-*tests indicated a lack of agreement between Zulu watch and Actiwatch 2 TST. Paired samples *t-*tests were also computed between the PSG and Actiwatch 2 measures of sleep; there was a lack of agreement between the TST and SE measures. The PSG and Actiwatch 2 measures of time of sleep onset and time of final awakening were in agreement.

**Figure 4:**
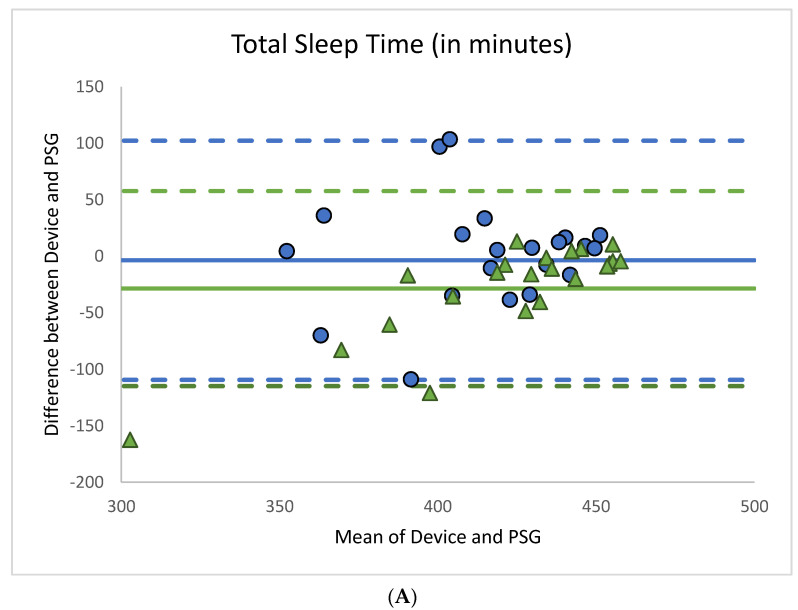

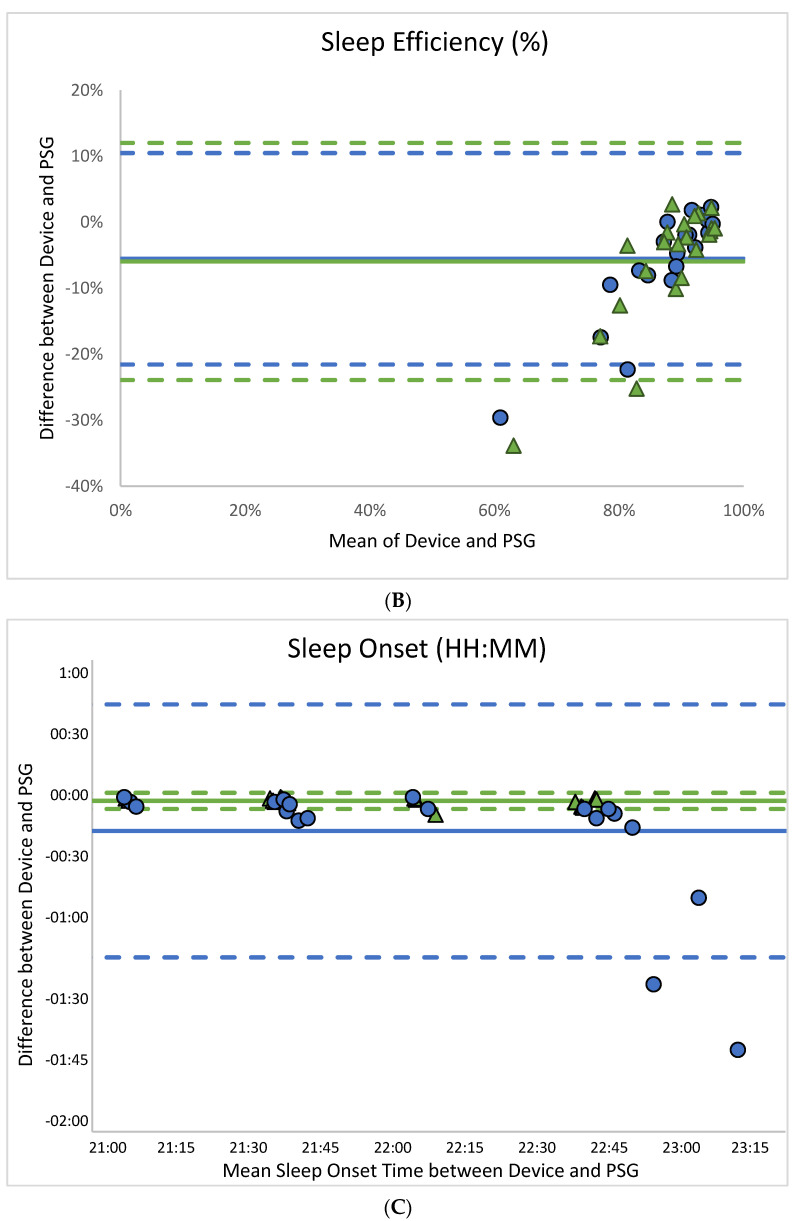

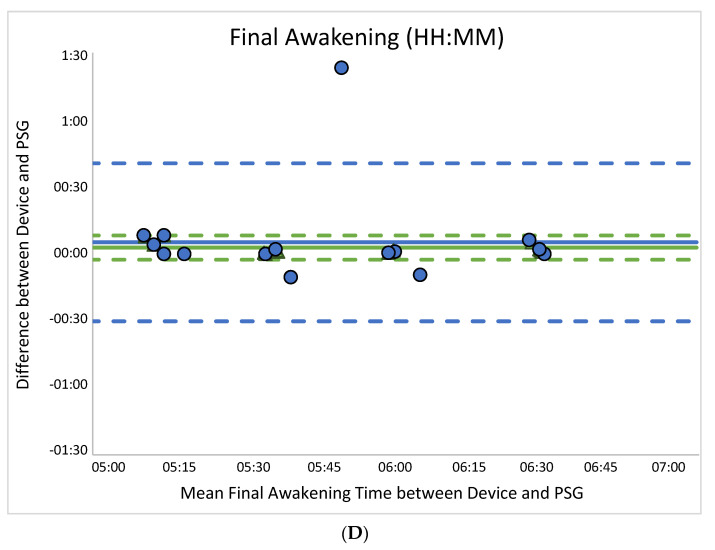
Bland–Altman plots of the differences (*y*-axis) between polysomnography (PSG) and the Zulu watch (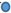) or PSG and the Actiwatch 2 (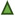) measures of sleep versus the mean of the two measurements (*x*-axis). Bias is represented by the solid blue line (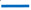) for the Zulu watch and the solid green line (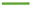) for the Actiwatch 2. Upper and lower limits of agreement (LOAs) are represented by the blue dashed lines (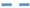) for the Zulu watch and green dashed lines (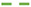) for the Actiwatch 2. Narrower LOAs indicate relatively less variability versus PSG than wider LOAs. Agreement is shown between measures for (**A**) total sleep time, (**B**) sleep efficiency, (**C**) time of sleep onset, and (**D**) time of final awakening. Time in bed (TIB) could not be visualized by the Bland–Altman plots because TIB was invariably set at 480 min for all participants across all nights for PSG and the Actiwatch 2.

The ICCs of the summary statistics across all three systems of measurement (PSG, Actiwatch 2, and Zulu watch) are summarized in Table 4. Based on the guidelines set forth by Koo and Li [26], ICCs for time of sleep onset and time of final awakening were good (0.75–0.90), indicating agreement between all three systems of measurement (PSG, Actiwatch 2, and Zulu watch). The ICCs were moderate for TST and SE (0.50–0.75) and poor for TIB (<0.50). Figure 4 summarizes the Bland–Altman plots of the mean difference between TST, SE, time of sleep onset, and time of final awakening, as measured by the Zulu watch or Actiwatch 2 compared against PSG.

### 3.4. Zulu Watch Extra Sleep Intervals

Across all participants and study days, Zulu watches logged 31 sleep intervals that occurred outside the PSG recording period. The interval median start times and duration for all extra intervals are summarized in Table 5. Intervals tended to be logged either in the early morning or in the late afternoon rather than midday. Median, rather than average, start times are reported to account for this bimodal distribution. Sixty-one percent (*n* = 19) of the extra intervals could be accounted for by instances when off-wrist detection indicated that the watch had been removed. Thirty-five percent (*n* = 11) occurred during in-laboratory study procedures. Seven of these in-laboratory extra intervals occurred when participants would have been stationary during morning cognitive performance tests at a computer, three instances occurred when participants would have been stationary during evening cognitive performance tests at a computer, and one instance occurred when the participant was stationary during the evening PSG electrode application. One logged interval could not be accounted for either by off-wrist detection or by laboratory procedures. The interval occurred in the morning (11:48) after study night 1 and lasted 30 min.

## 4. Discussion

While the true test of merit for the Zulu watch will be its performance in the field, laboratory validation is a crucial first step. Without validation testing, a sleep tracker lacks credibility in scientific or industrial research domains. Moreover, some common features of commercial sleep trackers, such as cloud-based sleep scoring or direct feedback to the user, may not be ideal for research. Sleep-tracking wearables serve an important role in the future of sleep research and sleep medicine [28,29] and have the capacity to surpass gold-standard actigraphy in terms of functionality and accuracy of sleep measurement in the real world. The Zulu watch is designed to fill a niche—sleep in the operational environment—and is capable of on-wrist sleep-wake determination, sleep-depth scoring, long battery life, and optional feedback functionality. The purpose of the current analyses was to evaluate the ability of the Zulu watch to measure sleep variables of interest against the gold-standards PSG and research-grade actigraphy. The Zulu watch showed mixed results on EBE sleep-wake and sleep-depth staging classifications, but it performed well compared with PSG and research-grade actigraphy on several key sleep summary metrics in a sample of healthy young adults.

The Zulu watch showed high accuracy for sleep-wake determination (>90%) compared with PSG and actigraphy. Sensitivity was also greater than 90% versus PSG and actigraphy. While specificity of the Zulu watch was low compared with the other measures, the specificity of the research-grade actigraph (Actiwatch 2) compared with PSG (21%) was comparable to the Zulu watch specificity (26%). Low specificity is an issue across research actigraphy devices and scoring algorithms [30,31], with longer epoch lengths relating to lower specificity [9]. It is possible that the Actiwatch 2 specificity was impacted by re-binning data from 30-s into 2-min epochs for comparison against Zulu watch data. Epochs longer than 60 s are not recommended when using Actiwatch devices and likely affect the specificity of the device [12,31,32]. Converting the epoch length to 2 min could have resulted in a lower specificity than found in other studies using 30- or 60-s actigraphy epoch lengths. Further, the high number of epochs scored as interrupted sleep by the Zulu watch may also contribute to its low specificity for wake versus PSG because many Zulu watch epochs scored as interrupted sleep may be scored as wake if using standard actigraphy algorithms. To better account for the duration and timing of wake or possible wake with the Zulu watch, perhaps the wake and interrupted sleep output metrics together could be a better reflection of sleep continuity through the night than wake alone.

Sleep-depth estimation by the Zulu watch could only be compared against PSG because sleep scoring is not a feature in actigraphy. The Zulu watch showed moderate accuracy for the estimation of sleep depth compared with PSG. Interestingly, the Zulu watch showed high sensitivity but low specificity for detecting deep sleep, and the inverse (high specificity and low sensitivity) for the detection of light or interrupted sleep compared with PSG. For deep sleep, the Zulu watch may be prone to Type I errors (i.e., falsely estimating an epoch to be deep sleep), but prone to Type II errors for light or interrupted sleep (falsely indicating that an epoch is not light or interrupted sleep). It is possible that the trade-off between sensitivity and specificity between sleep scores is due to the discrepancy between the Zulu watch’s sleep-scoring terminology versus PSG. The Zulu watch’s sleep scoring divides sleep periods into interrupted sleep, light sleep, and deep sleep, which do not directly correlate to the PSG scoring system. Previous studies have compared the commercial wearable or mobile app sleep scoring of light and deep sleep against PSG under the assumption that sleep stages N1 and N2 are comparable to light sleep, SWS is comparable to deep sleep, and REM is its own category [33,34,35,36]. However, the Zulu watch does not provide a separate category score for REM sleep; it provides the category of interrupted sleep, which has not been compared against PSG in previous studies. Without further guidance from the literature, we have attempted to achieve equivalence by recoding the PSG sleep stages into Zulu watch sleep scores by averaging the PSG 30-s epochs into 2-min bins. Interrupted sleep was scored as bins that contained a balanced mixture of sleep and wake epochs, as previously described. However, it should be noted that what constitutes interrupted sleep is open to interpretation and that the distinction between wake, interrupted sleep, and light sleep could differ on an EBE basis. Moreover, deep sleep could represent any stage of sleep or a combination of NREM and REM epochs, as indicated in Figure 1 by the arrows explaining the conversion between the PSG numerical scores and range of averages for the PSG scores. In a real-world environment, the Zulu watch estimation of sleep depth can help provide context on the quality of sleep opportunities that researchers may not otherwise be able to measure. However, Zulu watch sleep scores cannot and should not be considered equivalent to PSG sleep staging. Supplementary data sources, such as sleep diaries or daily schedules, as well as hand scoring EBE data, are recommended for any research or clinical use of actigraphy [9]. Supplementary measures may not always be practical to collect in operational research settings like the military, aviation, or shift work because they may increase the burden of data collection in a safety-sensitive environment [10]. This constitutes a limitation in the field of ecological sleep research technology in general. Researchers must use their best judgment when designing an operational research study design and when interpreting Zulu watch sleep scores in the context of their study.

Paired samples *t-*tests and ICCs showed good agreement between the Zulu watch, PSG, and actigraphy for time of sleep onset and time of final awakening. However, for analysis of both PSG and actigraphy, the scoring of these data was informed by the scheduled bed and wake times each night. This information is used to direct the RPSGTs and actigraphy algorithm where to begin looking for sleep onset and final awakening. On most nights, the Zulu watch determination of time of initial sleep onset and time of final awakening was comparable to both PSG and actigraphy without any input regarding bed or wake times.

There was moderate agreement between the Zulu watch, actigraphy, and PSG for TST and SE. Paired samples *t-*tests suggested that the limited agreement for TST and SE may in part be due to differences between actigraphy and PSG as well as differences related to the Zulu watch measures. Removing actigraphy or PSG measures did not influence the ICC results (see Supplementary Tables S1 and S2), which supports the finding that the Zulu watch has moderate agreement with either sleep measurement standards for TST or SE.

Agreement between Zulu and PSG or actigraphy for TIB was poor. One limitation to the analysis is that TIB was a constant measure (480 min) set by study staff across all nights of PSG and actigraphy data collection. Future evaluation of the Zulu watch would need greater variability in TIB to conduct more definitive comparisons. In the present study, the underestimation of TIB by the Zulu watch, as well as the device’s poor specificity for wake detection, most likely account for the device’s overestimation of SE. Another factor that may contribute to the Zulu watch’s underestimation of TIB is the lack of SOL estimation or time spent in bed after final awakening (snooze time) measurement by the Zulu watch. To explore this possibility, SOL and snooze time were estimated post hoc by subtracting the Zulu watch-determined time of sleep onset from the bed and wake times. Results were not statistically different between the Zulu watch and PSG (all *p* > 0.48). This finding indicates that a shorter TIB as measured by the Zulu watch is because the watch does not measure SOL or snooze time.

The Zulu watch has positive off-wrist detection so that periods when the watch is not being worn are not mistakenly scored as sleep, and it can measure sleep episodes as short as 20 min occurring at any time of the day. However, there were instances in the current study when the Zulu watch detected sleep intervals other than the study sleep period, even though participants were prohibited from napping and were closely monitored over the parts of the study while in the laboratory. Checking the sleep interval database against EBE data indicated that the majority (61%) of these recorded intervals registered as off-wrist periods. Further analyses are needed to determine how off-wrist periods could be misclassified as naps, and we recommend researchers using the Zulu watch verify naps against EBE data or a supplementary sleep diary. A design feature of the Zulu watch is collection and autonomous scoring of longitudinal sleep data in an operational environment. The Zulu watch can store scored sleep interval information for up to 80 sleep events but can only store 2-min EBE data for up to 7 consecutive days prior to extraction. Therefore, a limitation of the watch is that some off-wrist periods may be coded as naps. This discrepancy can be resolved by checking epoch logs, but only for data collected within the past week. We recommend that longitudinal data collection using the Zulu watch be extracted on a weekly basis to have complete EBE records. Moreover, the utility of the Zulu watch in this study was tested in a controlled laboratory environment in a sample of healthy sleepers. It remains to be seen if the watch functions similarly in operational environments, or for individuals with sleep disorders who may have more wake time and variability in their sleep patterns. As such, it is still recommended to ask participants to complete a sleep log to confirm sleep episodes or employ other methodology to confirm whether a sleep interval reflects true sleep versus a period of sedentary behavior or watch removal.

Our findings indicate that a device with single-sensor input and a sampling rate longer than 60 s (i.e., the Zulu watch) can detect sleep onset and final awakening with >80% agreement, and can differentiate sleep versus wake with >90% accuracy compared to PSG or research-grade actigraphy. These design choices may not seem preferable to a multi-sensor input and smartwatch-capable design, but they allow the Zulu watch to measure sleep without sacrificing battery life or requiring continuous synchronization with a cloud-based application. Extended battery life and on-wrist scoring are two aspects of device design that should be considered if the wearable is intended for operational or epidemiological data collection where compliance, continuous data collection, and data security are concerns. The next step after laboratory validation testing of the Zulu watch will be to test its utility in real-world operations, not only to measure sleep compared to field actigraphy or self-report, but also to see how the Zulu watch measurements of sleep or sleep depth can be used to inform assumptions about sleep behavior in military, aviation, or healthcare workers, in order to predict fatigue risk and on-the-job performance [37,38].

## 5. Conclusions

The longitudinal commercial sleep-tracking device, the Zulu watch, was evaluated against PSG and actigraphy as a sleep measurement device in a sample of healthy young adults over the course of a 3-day laboratory sleep study, with mixed results. The Zulu watch is not without its limitations, but showed high EBE accuracy and sensitivity for sleep and poor specificity for EBE detection of wake. The Zulu watch also showed high sensitivity for estimation of deep sleep (NREM + REM) compared with PSG, as well as moderate agreement for TST and SE. The Zulu watch was particularly good at determining the time of sleep onset and the time of final awakening. It is noteworthy that the Zulu watch can score sleep automatically on-wrist without requiring any intervention from the wearer or additional processing by a researcher or technologist. Moreover, the Zulu watch achieves this accuracy despite factors that could contribute to inaccuracy, such as long epoch lengths (2 min), on-wrist automatic scoring of sleep, and a lack of supplementary biometric sensor inputs like heart rate. These findings indicate that this monitoring system can reliably measure sleep using a minimalistic design. The Zulu watch represents an important step toward reliable, low-burden measurement of sleep in operational environments.

## Figures and Tables

**Table 1:** Accuracy, sensitivity, and specificity for sleep-wake determination.

	Accuracy	Sensitivity	Specificity
**Zulu compared with PSG**	90.28%	97.78%	25.68%
**Actiwatch 2 compared with PSG**	90.78%	98.86%	21.12%
**Zulu compared with Actiwatch 2**	94.24%	96.28%	32.94%

**Table 2:** Accuracy, sensitivity, and specificity of the Zulu sleep-depth scoring versus polysomnography.

	Accuracy	Sensitivity	Specificity
**Interrupted sleep**	87.17%	28.81%	92.12%
**Light sleep**	51.74%	10.73%	88.17%
**Deep sleep**	48.77%	84.16%	29.90%

**Table 3:** Comparison of Zulu watch sleep summary statistics against the PSG and actigraphy standards.

	**Zulu Mean**	**Standard**	**Standard Mean**	**Mean Difference**	**Paired Samples *t-*Test**
**TIB**	456′ ± 34′	PSG	480′ ± 0′	−24′	*t* = 3.43, *p* = 0.001 *
		Actigraphy	480′ ± 0′	−24′	*t* = 3.43, *p* = 0.001 *
**TST**	414′ ± 34′	PSG	408′ ± 57′	+6′	*t* = 0.45, *p* = 0.65
		Actigraphy	436′ ± 21′	−22′	*t* = 2.61, *p* = 0.01 *
**SE**	91% ± 3%	PSG	85% ± 12%	+6%	*t* = 2.29, *p* = 0.03 *
		Actigraphy	91% ± 4%	−0.1%	*t* = 0.06, *p* = 0.95
**Time of sleep onset**	22:05 ± 0:44′	PSG	22:02 ± 0:31′	+3′	*t* = 0.22, *p* = 0.83
		Actigraphy	21:53′ ± 0:31′	+12′	*t* = 1.03, *p* = 0.31
**Time of final awakening**	05:47 ± 0:28′	PSG	05:47 ± 0:33′	0′	*t* = 0.06, *p* = 0.95
		Actigraphy	05:49′ ± 0:29′	−2′	*t* = 0.20, *p* = 0.84
**Comparisons between PSG and Actigraphy**					
**TST**		PSG	408′ ± 57′	−28′	*t* = 2.23, *p* = 0.03 *
		Actigraphy	436′ ± 21′		
**SE**		PSG	85% ± 12%	+6%	*t* = 2.22, *p* = 0.03 *
		Actigraphy	91% ± 4%		
**Time of sleep onset**		PSG	22:02 ± 0:31′	−9′	*t* = 1.00, *p* = 0.32
		Actigraphy	21:53′ ± 0:31′		
**Time of final awakening**		PSG	05:47 ± 0:33′	+2′	*t* = 0.24, *p* = 0.81
		Actigraphy	05:49′ ± 0:29′		

PSG, polysomnography; SE, sleep efficiency; TIB, time in bed; TST, total sleep time; * *p* < 0.05.

**Table 4:** Intraclass correlation coefficients between Zulu watch, PSG, and actigraphy.

Summary Statistic	ICC	95% CI	Probability that ICC = 0 ^1^	Inter-Rater Reliability
**TIB**	0.46	0.09–0.83	*p* = 0.007 *	Poor
**TST**	0.54	0.26–0.76	*p* ≤ 0.001 **	Moderate
**SE**	0.60	0.33–0.83	*p* ≤ 0.001 **	Moderate
**Time of sleep onset**	0.87	0.72–0.96	*p* ≤ 0.001 **	Good
**Time of final awakening**	0.89	0.75–0.97	*p* ≤ 0.001 **	Good

CI, confidence interval; ICC, intraclass correlation coefficient; SE, sleep efficiency; TIB, time in bed; TST, total sleep time. ^1^ Significance for the probability that the coefficient is zero, implying no agreement; * *p* < 0.05; ** *p* ≤ 0.001.

**Table 5:** Zulu watch extra sleep intervals by time, duration, and reason for occurrence.

	Overall	Off-Wrist	Morning Laboratory Procedures	Evening Laboratory Procedures	Other
**Total number of intervals**	31	19	7	4	1
**Number per participant, mean (range)**	4 (2–8)	3 (0–5)	1 (0–2)	1 (0–1)	0 (0–1)
**Interval start time, median (range)**	09:56 (05:32–20:06)	10:10 (06:44–20:06)	07:44 (05:32–8:08)	17:52 (17:37–19:46)	11:48 (NA)
**Average duration, mean (range)**	52 min (20–166)	58 min (20–166)	51 min (26–122)	32 min (24–50)	30 min (NA)

## Data Availability

Restrictions apply to the availability of these data. Research data were obtained under a Cooperative Research and Development Agreement (CRADA) between the Institutes for Behavior Resources, Inc., and Naval Health Research Center.

## Supplementary Materials

The following are available online at https://www.mdpi.com/1424-8220/21/1/76/s1, Table S1: Intraclass Correlation Coefficients between Zulu Watch and PSG, Table S2: Intraclass Correlation Coefficients between Zulu Watch and Actigraphy.

## Disclaimer

This work was conducted (in part) under a Cooperative Research and Development Agreement (CRADA) between the Institutes for Behavior Resources, Inc., and Naval Health Research Center. Nothing in this document should be construed as an endorsement by the U.S. Navy. I am a military service member or employee of the U.S. Government. This work was prepared as part of my official duties. Title 17, U.S.C. §105 provides that copyright protection under this title is not available for any work of the U.S. Government. Title 17, U.S.C. §101 defines a U.S. Government work as work prepared by a military service member or employee of the U.S. Government as part of that person’s official duties. Report No. 20-97 was supported by the Office of Naval Research, Code 34, under work unit no. N1701. The views expressed in this article reflect the results of research conducted by the authors and do not necessarily reflect the official policy or position of the Department of the Navy, Department of Defense, nor the U.S. Government. The study protocol was approved by the Naval Health Research Center Institutional Review Board in compliance with all applicable federal regulations governing the protection of human subjects. Research data were derived from an approved Naval Health Research Center Institutional Review Board protocol, number NHRC.2017.0008.
