# Supplementary material for: Validation of Zulu Watch against Polysomnography and Actigraphy for On-Wrist Sleep-Wake Determination and Sleep-Depth Estimation
Source: Sensors (Basel). 2020 Dec 25;21(1):76. doi: 10.3390/s21010076 (PMC7796293; doi:10.3390/s21010076)
Supplement: Supplementary file 1 [file sensors-21-00076-s001.pdf]

sensors

MDPI

Supplementary Data

Supplementary Table S1. Intraclass Correlation Coefficients Between Zulu Watch and PSG

Summary Statistic	ICC	95% CI	Probability That IC=0 [1]	Inter-Rater Reliability
**TIB**	0.46	0.00–0.92	p=0.02*	Poor
**TST**	0.50	0.12–0.76	p=0.004*	Moderate
**SE**	0.60	0.26–0.87	p≤0.001**	Moderate
**Time of sleep onset**	0.84	0.64–0.95	p≤0.001**	Good
**Time of final awakening**	0.83	0.61–0.95	p≤0.001**	Good

CI, confidence interval; ICC, intraclass correlation coefficient; SE, sleep efficiency; TIB, time in bed; TST, total sleep time. [1] Significance for the probability that the coefficient is zero, implying no agreement; *p<0.05; **p≤0.001.

Supplementary Table S2. Intraclass Correlation Coefficients Between Zulu Watch and Actigraphy

Summary Statistic	ICC	95% CI	Probability That ICC=0 [1]	Inter-Rater Reliability
**TIB**	0.46	0.00–0.92	p=0.028*	Poor
**TST**	0.54	0.00–0.92	p=0.008*	Moderate
**SE**	0.51	0.06–0.81	p=0.011*	Moderate
**Time of sleep onset**	0.84	0.63–0.95	p≤0.001**	Good
**Time of final awakening**	0.84	0.62–0.96	p≤0.001**	Good

CI, confidence interval; ICC, intraclass correlation coefficient; SE, sleep efficiency; TIB, time in bed; TST, total sleep time.[1] Significance for the probability that the coefficient is zero, implying no agreement; *p<0.05; **p≤0.001.
